# Association of Fontan Pathophysiology With Plasma Bile Acids

**DOI:** 10.1016/j.jacadv.2024.101563

**Published:** 2025-01-18

**Authors:** Ashish H. Shah, Arun Surendran, Pedram Hassan-Tash, C. Nolan Turnbull, Nicole Johnston, David Goodlett, Jun Han, Robin A. Ducas, James W. Tam, Eberhard Renner, Todd A. Duhamel, Michel Aliani, Amir Ravandi, Richard A. Krasuski

**Affiliations:** a Department of Internal Medicine, Max Rady College of Medicine, University of Manitoba, Winnipeg, MB, Canada; b Department of Physiology and Pathophysiology, University of Manitoba, Winnipeg, MB, Canada; c Institute of Cardiovascular Sciences, Albrechtsen Research Center, St. Boniface Hospital, Winnipeg, MB, Canada; d Faculty of Kinesiology and Recreation Management, University of Manitoba, Winnipeg, MB, Canada; e Department of Biochemistry and Microbiology, University of Victoria, Victoria, BC, Canada; f Genome BC Proteomics Centre, University of Victoria, Victoria, BC, Canada; g Department of Food and Human Nutritional Sciences, University of Manitoba, Winnipeg, MB, Canada; h Division of Adult Congenital Heart Disease, Duke University Health System, Durham, NC, USA

**Keywords:** bile acids, Fontan circulation, metabolomics, pathophysiology

## Abstract

**Background:**

Patients with Fontan circulation are frail and experience multisystem dysfunction including impaired exercise capacity, low resting and exercise-augmented cardiac output, and progressive liver fibrosis. However, common underlying biochemical abnormalities or disease-specific biomarkers have not been well-described.

**Objectives:**

We wish to investigate Fontan and their matched healthy subjects using a nontargeted, followed by targeted metabolomic analysis.

**Methods:**

Patients with Fontan circulation were compared to age- and sex-matched healthy controls with regard to body composition, markers of frailty, cardiopulmonary exercise testing, and resting and exercise-augmented hemodynamics. Subsequently, the study participants underwent a nontargeted metabolomics assessment, followed by targeted plasma bile acid (BA) analysis.

**Results:**

Twenty Fontan patients (28.8 ± 9.8 years of age; 35% women) and 20 healthy controls (29.7 ± 6.0 years of age; 30% women) were enrolled. Fontan patients had significantly lower skeletal muscle mass, took longer to complete the 5 times sit-to-stand test; achieved lower %VO_2_ max, and had lower resting and postexercise hemodynamic parameters. Nontargeted metabolomics assessment demonstrated elevated BAs, oxylipins, and leucine metabolites in Fontan patients. Total BA as well as 17 BA components were markedly elevated in the Fontan patients. Selective BAs were negatively associated with age, degree of frailty, cardiopulmonary function, and hemodynamic parameters.

**Conclusions:**

Elevated BAs are associated with worsening Fontan physiology. These findings warrant further exploration.

Surgical correction of complex congenital abnormalities by creation of the Fontan circulation has saved the lives of countless newborns not amenable to biventricular repair.^1^ The Fontan correction separates the systemic and pulmonary circulation to prevent blood mixing and reduction of the systemic saturation, avoids volume overloading of the single ventricle, and facilitates survival to adulthood. Unfortunately, patients with this correction encounter chronic multisystemic complications, including liver, kidney, and musculoskeletal (sarcopenia and osteopenia); and have considerably reduced survival in comparison to healthy subjects.^2^ Moreover, patients with Fontan circulation develop progressive liver fibrosis that can progress to cirrhosis. Heart or heart-liver transplant is the only curative option, though very few patients meet the required stringent criteria. Despite Fontan associated multisystem dysfunction, underlying biochemical abnormalities, or Fontan-specific biomarkers have not been well-described.

Nontargeted metabolomic analysis exploring a broad spectrum of metabolites without prior selection can unravel novel biomarkers and metabolic pathways associated with any pathological state. Once potential targets are identified, targeted metabolomic analysis allows precise quantification of specific metabolites of interest. Subsequently, these biomarkers need correlating with clinical and investigational parameters of interest to explore their association. This two-step approach is a well-established method in understanding metabolic alterations, and discovering disease-specific, outcome-associated biomarkers.^3^ Such knowledge may enhance earlier diagnosis, improve risk stratification, and provide timely treatment strategies that may improve clinical outcome. We decided to utilize such an approach investigating patients with Fontan circulation.

## Materials and methods

### Study participants

Twenty adult (>18 years of age) patients with Fontan circulation and 20 age- and sex-matched healthy (no history of congenital heart disease or systemic disorders) controls were prospectively recruited. Exclusion criteria for Fontan patients included NYHA functional class IV symptoms, major intellectual or physical disability interfering with ability to understand or participate in the study and recent or prior referral for heart transplantation. Informed consent was obtained from all participants and the local Research and Ethics Board approved the study.

### Body composition and frailty assessment

Each study participant underwent body composition assessment using the InBody 570 Body Composition Analyzer (InBody Co Ltd), which utilizes bioelectrical impedance analysis technology. Measured parameters included skeletal muscle mass (SMM) (total weight of skeletal muscle), lean body mass (total body weight − fat weight), and dry lean mass (total body weight − water and fat weight). The accuracy of this technology is comparable to dual x-ray absorptiometry scanning,^4^ and its utility has been described previously.^5^ Study participants were further investigated by 5-m gait (seconds), 5 times sit-stand test (5XSST, measured in seconds), and handgrip strength measurement (kg).

### Cardiopulmonary exercise testing

Participants underwent stress testing using a modified, symptom-limited Balke protocol. A progressive ramp exercise test was performed using a treadmill, a nonrebreathing mask, and a gas analyzer (Vyntus Metabolic cart [CMO17695]; Vyaire Medical). After 2 minutes of warm-up, the exercise intensity was increased every 2 minutes incrementally to the point of exhaustion. Subjective level of exertion, reason for stoppage, and VO_2max_ were recorded.

### Noninvasive hemodynamic evaluation

Resting and post-cardiopulmonary exercise testing (CPET) hemodynamic parameters were measured using the Non-Invasive Cardiac System (NICaS) (NI medical), a whole body impedance technology, as previously described.^6^ Study participants were placed in a relaxing supine position for 10 minutes prior to resting hemodynamic evaluation. Two NICaS tetrapolar electrodes were applied in a left-wrist and right ankle configuration. Once initiated, the NICaS measured hemodynamic parameters every 20 seconds. After 5 consistent measurements in the supine position, hemodynamic parameters were documented. Study participants were made supine immediately post-CPET, and hemodynamic parameter measurements were repeated.

### Nontargeted plasma metabolomics

Fasting blood samples were collected from each study participant. Plasma was separated after centrifugation at 4,000 rpm for 10 minutes at 4 °C temperature and stored in −80 °C until analysis. One hundred microliter of plasma and 200 μL of acetonitrile were mixed, centrifuged, and supernatant separated for analysis using a LC-MS method as described previously^7^ (Supplemental Appendix).

### Plasma bile acid analysis

BA analysis was performed using ultrahigh performance liquid chromatography coupled to a tandem mass spectrometer (UPLC-MS/MS) on a Sciex QTRAP 6500 plus mass spectrometer operated in the negative-ion multiple-monitoring reaction (MRM) mode, using the LC and MS parameters as published previously.^8^^,^^9^ The UPLC-MRM/MS data set were recorded using the Sciex Analyst Software and processed using the Sciex MultiQuant Software for peak detection and peak integration. Concentrations of total and 75 measurable BA components in the plasma samples were calculated with the analyte-to-internal standard peak area ratios measured in each sample solution to interpolate the constructed linear-regression, internal standard-calibration curves of individual BA standards in an appropriate concentration range for each analyte. The study flow is described in Central Illustration.

**Central Illustration:**
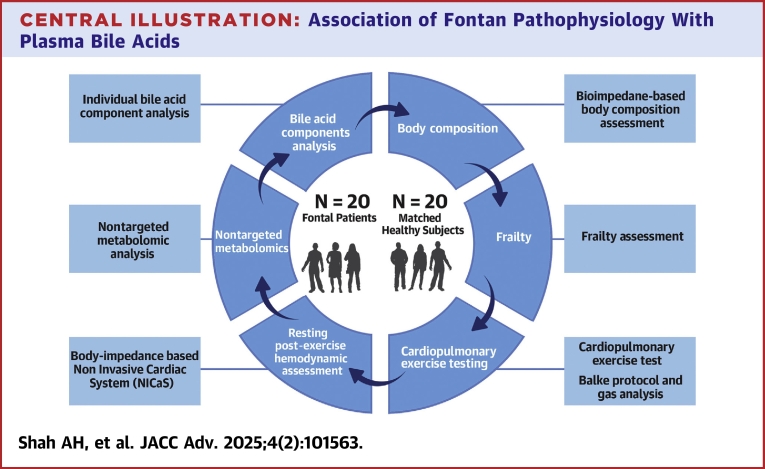
Association of Fontan Pathophysiology With Plasma Bile Acids Assessment of Fontan patients and healthy controls from time of recruitment. This included body composition and frailty assessment, cardiopulmonary exercise testing, noninvasive hemodynamic assessment (pre- and post-exercise), nontargeted metabolomics, plasma bile acid measurement and correlation of total and individual bile acid components with other parameters.

### Statistical analysis

Data from Fontan patients were compared to healthy subjects using unpaired *t*-tests, whereas pre- and post-exercise changes were compared using paired *t*-tests. F-test was utilized to determine whether homogeneity in terms of variance might be assumed or not. Values are reported as mean ± SD, unless otherwise stated. A *P* value of <0.05 was considered significant. Heat maps were used to visualize the bile acid (BA) concentration differences between Fontan patients and controls, where the concentrations were standardized using log transformation. To determine which BAs were significantly different in Fontan patients from controls, multiple *t*-tests were applied. Finally, we examined how the body composition indicators, cardiopulmonary exercise test results, and hemodynamic variables correlated with individual BA concentrations. Pearson correlation coefficients were calculated to explore the existence of a linear relationship between pairs of variables. All statistical analyses were performed using R, version 3.6.1 (The R Foundation, Vienna, Austria). Principal component analysis (PCA) and partial least squares discriminant analysis (PLS-DA) were carried out using MetaboAnalyst (v5.0).

## Results

### Demographics, body composition, degree of frailty, and Fontan type

Twenty Fontan patients (mean age 28.8 ± 9.8 years; 35% women) and 20 age- and sex-matched healthy subjects (29.7 ± 6.0 years of age; 30% women) were enrolled. Despite having similar heights, Fontan patients trended toward lower weights (69.7 ± 14.5 kg vs 77.6 ± 13.8 kg, *P* = 0.08), mainly due to significantly less SMM (27.9 ± 6.3 kg vs 34.3 ± 7.8 kg, *P* = 0.007). Similarly, Fontan patients had lower lean body mass (total weight − fat mass) (50.8 ± 10.0 kg vs 60.4 ± 12.8 kg, *P* = 0.0118) and dry lean mass (total weight − water and fat mass), (13.5 ± 2.8 kg vs 16.2 ± 3.5 kg, *P* = 0.0092). Fontan patients also took longer to complete the 5XSST (9.6 ± 3.1 seconds vs 5.7 ± 1.3 seconds, P=<0.0001) and had lower handgrip strength (35.6 ± 11.9 kg vs 42.6 ± 10.1 kg, *P* = 0.054), but could walk a 5-m distance at a similar pace to healthy subjects. Study participants’ demographics, body composition, frailty assessment, Fontan type and age since the Fontan completion is described in Table 1.

**Table 1:** Demographics, Body Composition, Frailty, and Fontan Type

Parameter	Fontan (n = 20)	Healthy (n = 20)	*P* Value
Males	13 (65%)	14 (70%)	1.0000
Age (y)	28.8 ± 9.8	29.7 ± 6.0	0.7132
Height (cm)	169.7 ± 9.5	171.9 ± 8.8	0.4494
Weight (kg)	69.7 ± 14.5	77.6 ± 13.8	0.0836
Body mass index (kg/m^2^)	24.2 ± 4.5	26.1 ± 3.2	0.1183
Body fat mass (kg)	19.7 ± 9.0	17.4 ± 6.5	0.3728
SMM (kg)	27.9 ± 6.3	34.3 ± 7.8	0.0070^b^
Lean body mass (kg)	50.8 ± 10.0	60.4 ± 12.8	0.0118^a^
Dry lean mass (kg)	13.5 ± 2.8	16.2 ± 3.5	0.0092^b^
% body fat	27.5 ± 9.8	22.7 ± 8.3	0.1010
Left arm (kg)	2.6 ± 0.7	3.4 ± 1.0	0.0052^b^
Left leg (kg)	7.8 ± 1.9	8.8 ± 1.7	0.0686
Trunk (kg)	22.3 ± 4.4	26.9 ± 5.6	0.0067^b^
Right arm (kg)	2.6 ± 0.7	3.5 ± 1.0	0.0046^b^
Right leg (kg)	7.8 ± 1.8	8.9 ± 1.7	0.0597
Basal metabolic rate (kcal)	1,456 ± 229	1,676 ± 277	0.0095^b^
5 x - Sit-to-Rise Test (seconds)	9.6 ± 3.1	5.7 ± 1.3	<0.0001^c^
Hand-grip (kg)	35.6 ± 11.9	42.6 ± 10.1	0.054
5-m walk (seconds)	3.9 ± 0.5	4.1 ± 0.6	0.118

Fontan Type and Time Since Completion	
Extracardiac	14
Lateral tunnel	4
Bjork-type repair	1
Classic Fontan (atriopulmonary connection)	1
Years since completion	22 ± 6

Values are n (%) or mean ± SD.

Lean body mass = total weight–fat mass; dry lean mass = total weight–water and fat mass.

SMM = skeletal muscle mass.

a *P* < 0.05.

b *P* < 0.01.

c *P* < 0.001.

### Pulmonary function testing and cardiopulmonary exercise studies

Fontan patients had significantly impaired pulmonary function test parameters compared to healthy subjects, namely forced vital capacity (FVC) (3.5 ± 11 L vs 5.7 ± 1.7 L, *P* = 0.0002), FVC (% predicted) (82.9 ± 19.2 vs 120 ± 20.2%, *P* < 0.0001), forced expiratory volume in 1 second (FEV1) (2.9 ± 0.9 L vs 4.5 ± 1.1 L, *P* < 0.0001), and FEV1 (%predicted) (76.6% ± 15.8% vs 113.2% ± 16.7%, *P* < 0.0001). However, absolute FEV1/FVC and % predicted values did not appear different. Fontan patients were able to exercise less than controls (14.1 ± 5.1 minutes vs 17.6 ± 4.4 minutes, *P* = 0.02). Peak oxygen consumption (VO_2max_) during exercise in Fontan patients was considerably less (23.7 ± 5.6 mL/kg/min [64.8% ± 14.0% expected]) when compared to controls (43.9 ± 8.6 mL/kg/min [124.3% ± 23.2% expected]), *P* < 0.0001 for both comparisons. Similarly, Fontan patients had lesser chronotropic response (peak heart 154 ± 26 beats/min vs 185 ± 11 beats/min, *P* < 0.0001) and a higher minute ventilation − carbon dioxide production relationship (VE/VCO_2_) slope (39.3 ± 4.8 vs 32.0 ± 3.6, *P* < 0.0001). Pulmonary function and CPET results are detailed in Table 2.

**Table 2:** Pulmonary Function, Cardiopulmonary Exercise Test Data, and Hemodynamic Assessment of Fontan Patients and Healthy Controls

	Fontan (n = 20)	Control (n = 20)	*P* Value
Spirometry results			
FVC	3.7 ± 1.1	5.7 ± 1.7	0.0002^c^
FVC % predicted	82.9 ± 19.2	120 ± 20.2	<0.0001^c^
FEV1	2.9 ± 0.9	4.5 ± 1.1	<0.0001^c^
FEV1 (% predicted)	76.6 ± 15.8	113.2 ± 16.7	<0.0001^c^
FEV1/FVC %	79.8 ± 8.9	79.4 ± 5	0.8824
FEV1/FVC (% predicted)	94.6 ± 10.2	94.4 ± 5.8	0.9552
Cardiopulmonary exercise test			
Treadmill test phase time achieved (min)	14.1 ± 5.1	17.6 ± 4.4	0.0246^a^
METS achieved	6.8 ± 1.6	12.3 ± 2.6	<0.0001^d^
VO_2max_ (mL/kg/min)	23.7 ± 5.6	43.9 ± 8.6	<0.0001^c^
VO_2max_ (% predicted)	64.8 ± 14.0	124.3 ± 23.2	<0.0001^c^
VE/VCO_2_	39.3 ± 4.8	32.0 ± 3.6	<0.0001^c^
RER	1.1 ± 0.08	1.18 ± 0.07	<0.0001^c^
Peak HR (beats/min)	154 ± 26	185 ± 11	<0.0001^c^
Hemodynamics - rest			
SI	27.6 ± 7.9	45.5 ± 7.4	<0.0001^c^
Cardiac index	2.1 ± 0.7	3 ± 0.5	0.0002^c^
CO	3.9 ± 1.6	5.7 ± 1.1	0.0003^c^
CPI	0.4 ± 0.2	0.6 ± 0.1	0.0001^c^
CPO	0.7 ± 0.3	1.2 ± 0.3	0.0002^c^
TPRI	3526.7 ± 1,306.9	2,517.7 ± 464.1	0.0034^b^
Hemodynamics - post-exercise			
SI	27.9 ± 7.7	47 ± 8	<0.0001^c^
SV	50.9 ± 15.7	90.3 ± 21	<0.0001^c^
Cardiac index	2.8 ± 0.8	4.9 ± 1.1	<0.0001^c^
CO	5.1 ± 1.7	9.3 ± 2.3	<0.0001^c^
CPI	0.6 ± 0.2	1.2 ± 0.3	<0.0001^c^
CPO	1.0 ± 0.4	2.3 ± 0.6	<0.0001^c^
TPRI	2,863.2 ± 978.2	1894.4 ± 475.3	0.0005^c^

Values are mean ± SD.

CO = cardiac output; CPI = cardiac power index; CPO = cardiac power output; FEV1 = forced expiratory volume in 1 second; FVC = forced vital capacity; METS = metabolic equivalents; RER = respiratory exchange ratio; SI = stroke index; TPRI = total peripheral vascular resistance; SV = stroke volume; VE/VCO_2_ = minute ventilation − carbon dioxide production relationship.

a *P* < 0.05.

b *P* < 0.01.

c *P* < 0.001.

d *P* < 0.0001.

### Hemodynamics at rest and post-exercise

At rest, Fontan patients had significantly lower stroke index (SI) (27.6 ± 7.9 vs 45.5 ± 7.4 mL/m^2^, *P* < 0.0001), cardiac index (CI) (2.1 ± 0.7 vs 3.0 ± 0.5 L/min/m^2^, *P* = 0.0002), and cardiac power index (CPI) (0.4 ± 0.2 vs 0.6 ± 0.1 W/m^2^, *P* = 0.0001), but higher total peripheral vascular resistance (3,527 ± 1,307 vs 2,518 ± 464 dyn/s/cm^5^/m^2^, *P* = 0.003). Hemodynamic assessment immediately following CPET also demonstrated significantly lower SI (27.9 ± 7.7 vs 47 ± 8 mL/m^2^, *P* < 0.0001), cardiac index (2.8 ± 0.8 vs 4.9 ± 1.1 L/min/m^2^, *P* < 0.0001), and CPI (Fontan: 0.6 ± 0.2 vs healthy: 1.2 ± 0.3 W/m^2^, *P* < 0.0001) in Fontan patients, but higher total peripheral vascular resistance (2,863 ± 978 vs 1,894 ± 475 dyn/s/cm^5^/m^2^, *P* = 0.0005). The ΔCI and ΔCPI were flat in Fontan patients compared to controls. Hemodynamic parameters are listed in Table 2.

### Nontargeted metabolomics evaluation

**Figure 1:**
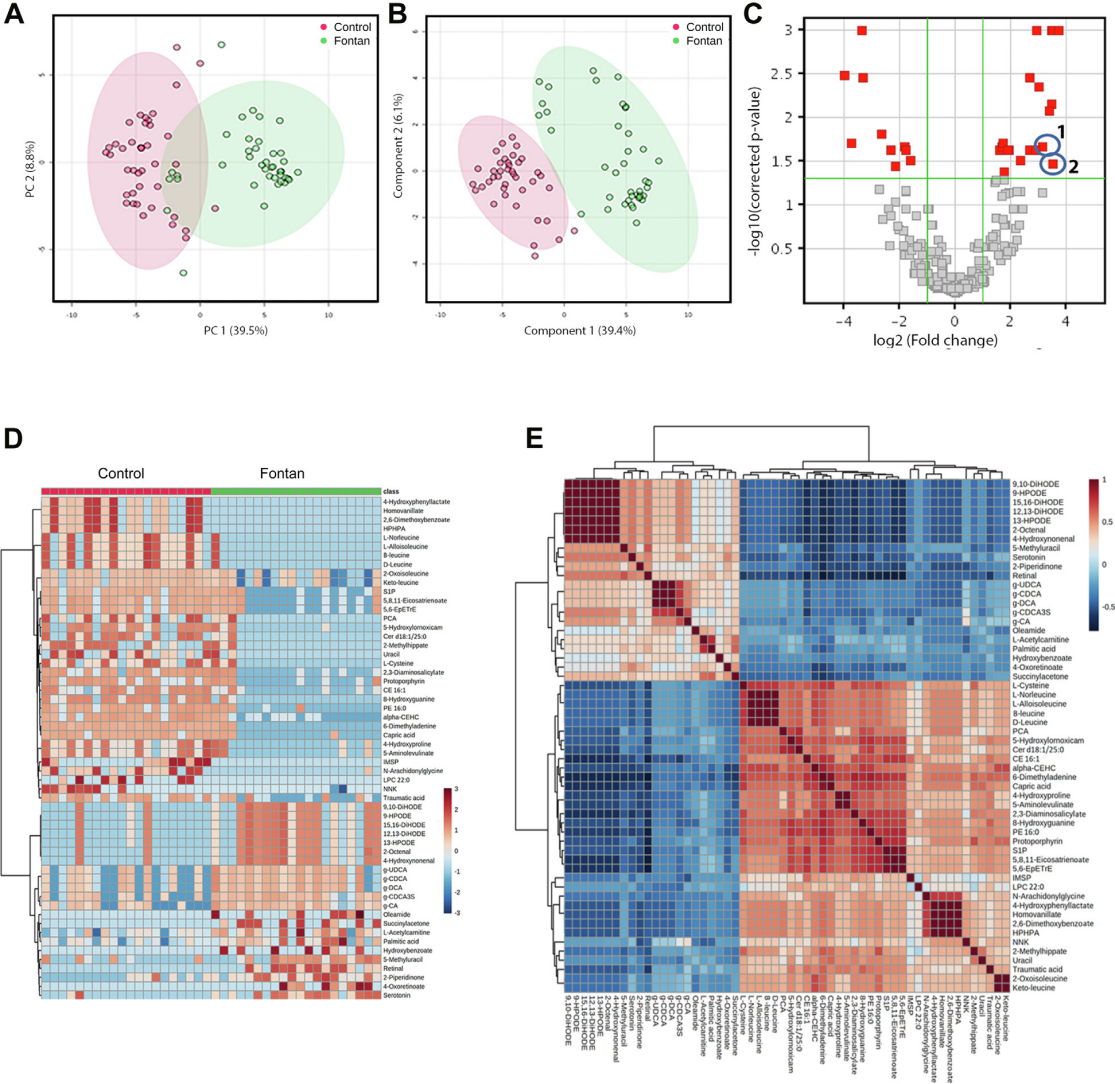
Metabolomics of Fontan Patients and Healthy Controls Principal component analysis scores plot (A), and partial least square-discriminant analysis scores plot (B) of fontan patients compared to healthy controls (B). (C) Volcano plot of metabolomics data. X- and Y-axis present the log 2 (ratio) and −log10 (corrected *P* value), respectively. Red color denotes the metabolites with significant difference (*P* < 0.05 and a fold change >4). (D) Clustered Heatmap depicting differential abundance of metabolites between Fontan patients and healthy controls. each column represents a sample and each row represents a metabolite. The red and blue colors correspond to high or low metabolite levels, respectively. (E) Correlation Heatmap depicting metabolite-metabolite correlations (Pearson correlation) of 58 metabolites that were significantly different between Fontan patients and healthy controls.

Multivariate analyses (PCA and PLS-DA) were used to visualize the variation in metabolic characteristics. The unsupervised PCA (Figure 1A) demonstrated good separation between the normalized data for the two groups. The data sets were further analyzed using the supervised PLS-DA approach (Figure 1B), which also displayed clear separation between the two groups. Overall, these results demonstrated marked differences in the metabolic characteristics of Fontan patients compared to controls. A volcano plot (Figure 1C) was then built to identify the most discriminatory metabolic features between Fontans and controls. An adjusted *P* value <0.05 and a fold change >4 were set as cutoff values. The discriminatory features are highlighted in red. Interestingly, two BAs (1-glycochenodeoxycholate-3-sulfate [FC = 11.4] and 2-glycochenodeoxycholic acid 3-glucuronide [FC = 8.8]) exhibited large differences between groups.

To understand the clustering relationship and relative abundance of differential metabolites, a hierarchical clustering heatmap was built using the 58 metabolites that were significantly different between Fontan patients and healthy controls after analysis of variance testing with an adjusted *P* < 0.05 (Figure 1D). Noteworthy is the fact that BAs formed a tight clustering in the heatmap, thereby revealing the similarities in their action. The abundance of the BAs was higher in Fontan patients than controls. To get an overall impression, the pairwise correlations (Pearson correlation) among all significant metabolites are shown as a correlation heatmap (Figure 1E). There was a cluster of positively correlated BAs (glycoursodeoxycholic acid, glycochenodeoxycholic acid, glycodeoxycholic acid) shown as a unique fingerprint in the heatmap.

### BA analysis

**Figure 2:**
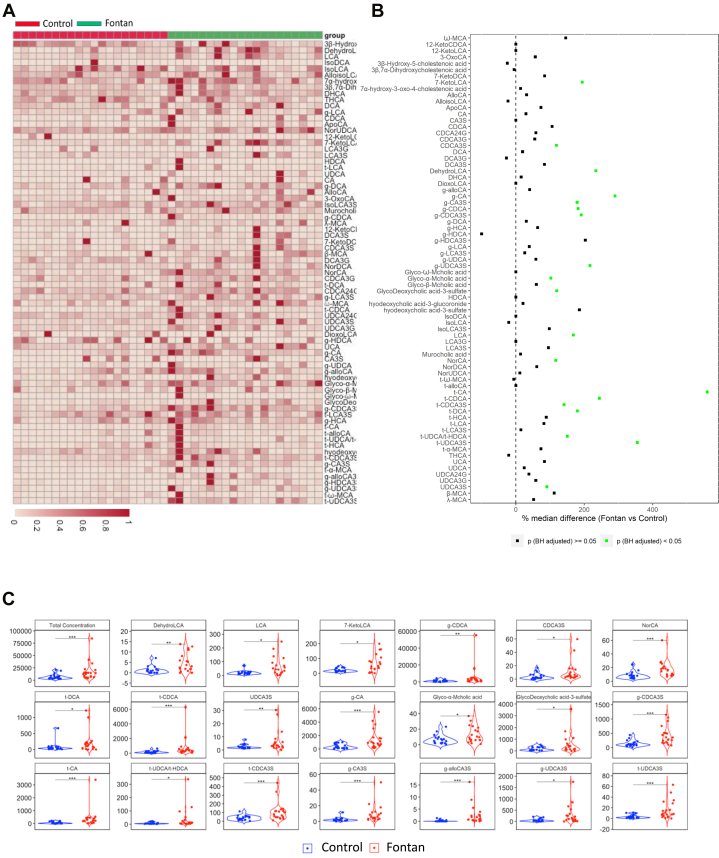
Bile Acids Analysis, Their Distribution on the Spectrum of Hydrophobicity–Hydrophilicity and Individual Elevated BA in Fontan Patients and Healthy Controls (A) Seventy-five bile acids measured in each Fontan patient and healthy control (red–healthy subject; green–Fontan patient). (B) Bile acid concentration differences between Fontan patients and healthy subjects; bile acids are described by their spectrum of Hydrophobicity–Hydrophilicity. Significantly elevated bile acids in Fontan patients are described in green color. (C) Total (75) and the 17 significantly elevated bile acids in fontan patients.

Total BA concentration was significantly elevated in Fontan patients compared to healthy controls (17,601 ± 18,084 vs 7,163 ± 5,136 nM; *P* = 0.02). Seventy-five individual BA components were further analyzed (Figure 2A), of which 17 were markedly elevated in Fontan patients. The distribution of significantly elevated BAs across the spectrum of hydrophobicity–hydrophilicity is illustrated in Figure 2B, whereas the significantly elevated BAs are described in Figure 2C and Supplemental Table 1. The majority of elevated BA were secondary and were scattered across the spectrum of hydrophobicity–hydrophilicity.

### Correlation of investigations

**Figure 3:**
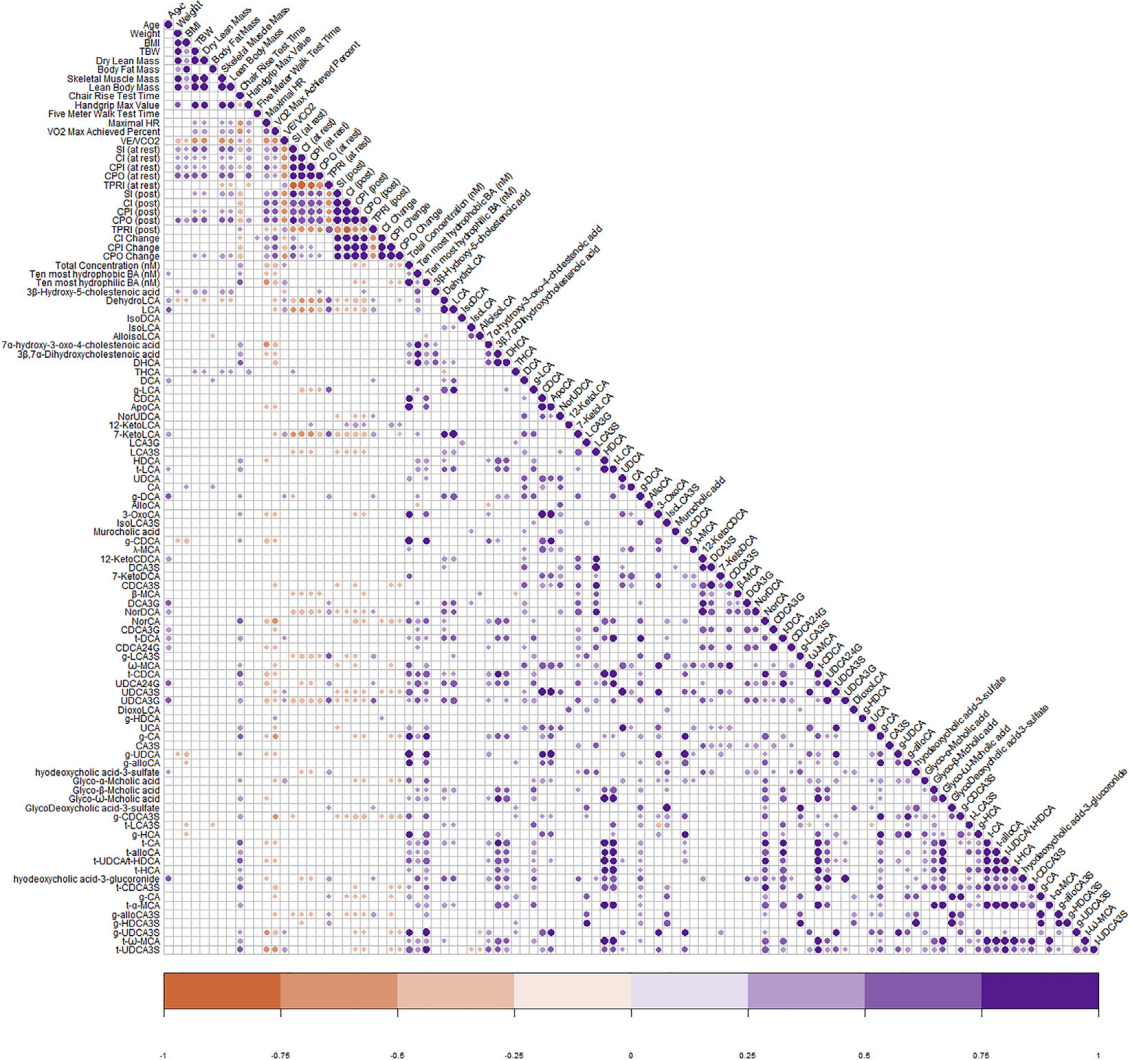
Bile Acids and Their Correlation With Demographics, Frailty, Exercise Capacity, and Hemodynamic Parameters The relationship between demographic, frailty, exercise capacity, hemodynamic parameters, and the total as well as individual 75 bile acids. Presence of dot signifies a significant correlation. purple color describes a positive relationship, whereas orange color describes a negative association.

Pearson correlation between demographics, body composition, frailty, cardiopulmonary exercise, and hemodynamic parameters with BA is shown in Figure 3. Increasing age was associated with reduced SMM and worsened exercise capacity and hemodynamic parameters. The 5XSST was the only frailty measurement noted to be significantly impaired, and correlated with age, body composition, and hemodynamic parameters, as well as various BAs. SI and CPI also negatively correlated with age, body composition, degree of frailty, and CPET.

**Figure 4:**
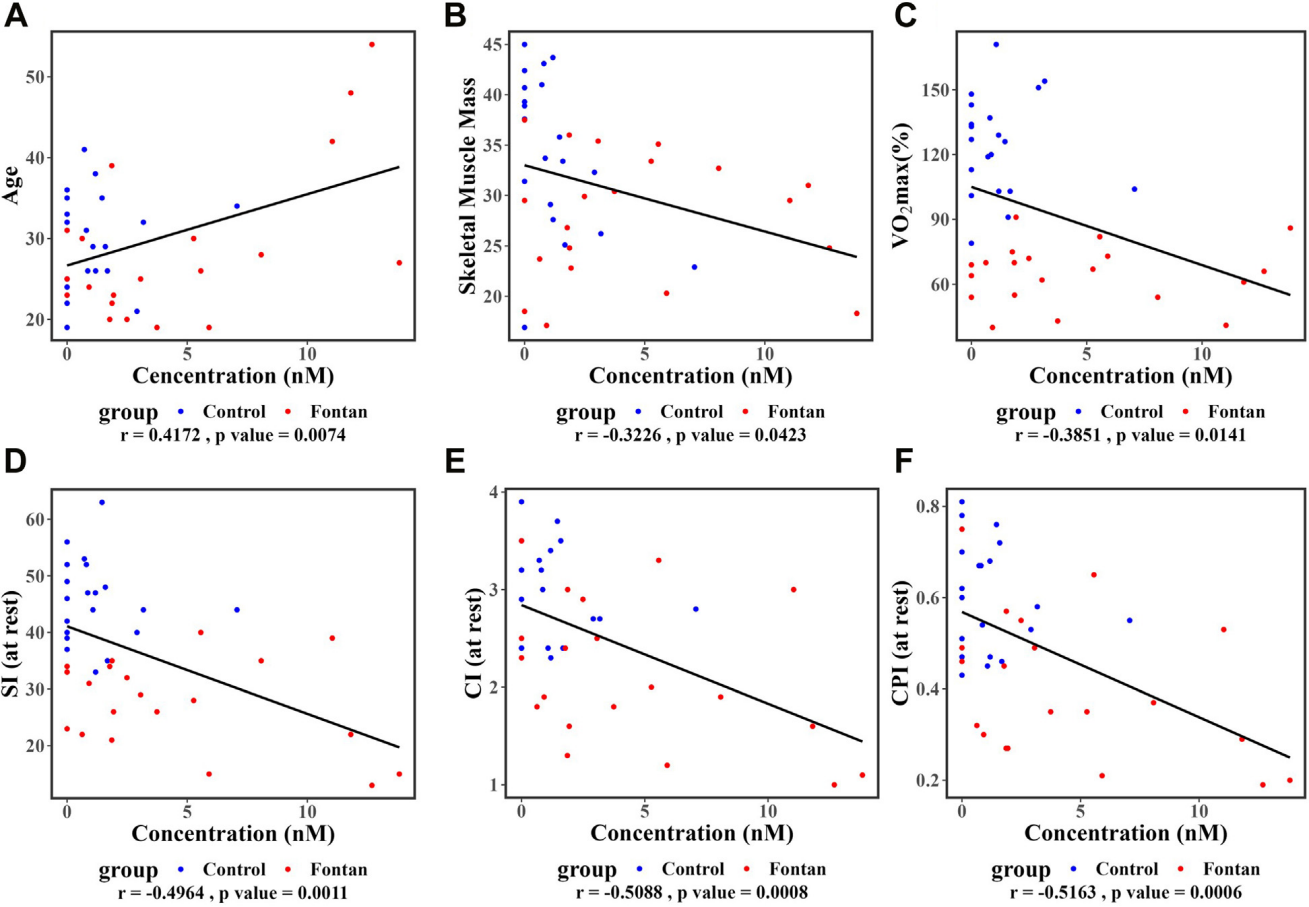
DehydroLithocholic Acid Level and Its Correlation With Age, Body Composition, Exercise Capacity, and Hemodynamic Parameters The relationship between dehydro lithocholic acid concentration with (A) age, (B) skeletal muscle mass, (C) VO_2max_ (%), (D) SI (at rest), (E) CI (at rest), and (F) CPI (at rest). Blue color represent Fontan patient, and red color represent heathy subject. CI = cardiac index; CPI = cardiac power index; SI = stroke index; VO_2max_ = maximal oxygen consumption.

Through further evaluation of the 17 significantly elevated BA in Fontan patients with respect to other parameters including age, body composition, degree of frailty, CPET performance, and hemodynamic parameters, we identified that lithocholic acid (LCA), dehydro-LCA, and 7-keto-LCA correlated negatively with many CPET and hemodynamic parameters. The correlation of dehydro-LCA with various clinical and biochemical parameters is described in Figure 4.

## Discussion

This study provides an in-depth biochemical characterization of adult patients with a Fontan circulation. Fontan patients were noted to have: 1) age-associated low SMM, which is disproportionate to body mass index (BMI); 2) frailty, best assessed by 5XSST in comparison to hand-grip strength or 5 m walk test; 3) low %VO_2max_ and elevated VE/VCO_2_ slope on CPET; 4) low resting and exercise-augmented SI, cardiac index, and CPI, and most importantly; 5) elevated BAs, which are associated with increased frailty, reduced exercise capacity, and worsened hemodynamic parameters.

Recent publication has described elevated BAs in younger Fontan patients with heart failure.^10^ Our findings are unique in the terms that we have demonstrated elevated BAs and their associations with various clinical/investigational parameters of interest. In addition to their well-described role in fat metabolism and fat-soluble vitamin absorption in the small intestine, our understanding into the functionality of BAs has significantly improved over the last few years. Despite the presence of multi-organ dysfunction in many Fontan patients, no Fontan-specific biomarkers have previously been identified to quantify underlying organ abnormalities or offer outcome prognostication. Given these circumstances, instead of choosing biomarkers defined for other disease states (such as cystatin C, creatinine, or alpha-fetoprotein), we chose to investigate Fontan patients using nontargeted metabolomics followed by a targeted biomarker evaluation. Although we also observed markedly elevated oxylipin leucine metabolites in Fontan patients, in the absence of in-depth understanding with these metabolites and their role in cardiovascular pathologies, we chose to instead proceed with detailed BA analysis, as Fontan patients are also characterized by progressive liver fibrosis and elevated BA appeared to be a unifying signaling molecule in this patient population.

### Body composition: frailty and elevated BAs

Fontan patients are characterized by pronounced lower SMM and excess adiposity; hence, the BMI may not be precisely representative of their body composition.^11^ Higher SMM and lean body mass is associated with better %VO_2max_ achieved, a marker of healthy cardiovascular status.^11^^,^^12^ Observed discrepancy between BMI and SMM raises a concern with the routine use of BMI as a clinical tool in this patient population. Moreover, 5XSST better identified frailty among Fontan patients compared to handgrip or 5-m walk test. The sit-stand test requires interplay between various long muscles across the lower torso and thigh; longer duration for completing this simple test may be representative of significant muscle atrophy and sarcopenia. Observed wasting of the psoas muscle at the level of the L3 vertebra is associated with adverse outcome in patients presenting with acute coronary syndrome or severe aortic stenosis.^13^^,^^14^ However, the utility of such measurement in Fontan patients has not been previously reported.

It is important to delineate cause from effect among these changes in cardiac output, frailty, and elevated BAs. Muscle wasting is also observed in patients with myocardial or valvular pathologies complicated by low cardiac output states, a condition often referred to as “cardiac cachexia.”^15^ The observed cachexia could be due to persistently low cardiac output, physical deconditioning, elevated BAs, or a combination of all these elements.^16^^,^^17^ Similar to patients with Fontan-associated liver disease, patients with other liver pathologies have exaggerated SMM wasting.^16^ BA-mediated skeletal muscle atrophy occurs through its direct interaction with the muscle membrane receptor, Takeda G protein-coupled receptor 5, leading to excess oxidative stress.^18^^,^^19^ The majority of elevated BAs observed in Fontan patients were secondary; only the gut microbiome interacts with primary BAs to convert them into secondary BAs. Antibiotic treatment-mediated gut microbiome depletion in the mouse led to altered BA composition and subsequently reduced SMM.^20^ Interestingly, patients with low cardiac output (CO) have been noted to have altered gut microbiome composition.^21^ Similarly, low CO in Fontan patients may adversely influence gut microbiome volume and/or composition. Additionally, excess body-fat (adiposity) is reported to be associated with adverse outcomes in patients with Fontan circulation.^22^ Measures of these parameters should be incorporated into the routine clinical care of Fontan patients to objectively define any deterioration.

### Exercise capacity and hemodynamic response

CPET is commonly utilized to evaluate Fontan patients and low %VO_2max_ in these patients is an “acceptable norm.” Interval decline in peak oxygen consumption (>3%/year) is associated with adverse clinical events, need for heart transplantation and mortality.^23^^,^^24^ Fontan patients also have reduced chronotropic competence (maximal heart rate at the peak exercise).^25^ Although patients can generally exercise longer after Fontan completion, their overall exercise capacity remains markedly lower than matched controls.^25^ The observed association between resting CO and %VO_2max_ achieved on CPET suggests that noninvasively obtained hemodynamic parameters in the outpatient setting can provide important prognostic information.^26^ Fontan patients have low SI at rest and a blunted peak heart rate, both of which influence ability to augment CO at peak exercise. In patients with biventricular circulation, low stroke volume has been associated with new-onset heart failure, independent of ventricular geometry, systolic function, or other major risk factors.^27^ Lower peak heart rate achieved during exercise has also been associated with excess mortality.^23^^,^^24^^,^^28^

### Bile acids and fontan circulation

BAs are the end products of cholesterol catabolism and are synthesized in the pericentral hepatocytes of the liver. Primary BAs (cholic acid and chenodeoxycholic acid) are released into gut. The gut microbiome converts these primary BAs into secondary BAs that can then be absorbed from the intestine and return to liver as a part of enterohepatic circulation. In addition to their role in fat metabolism, emerging evidence supports BAs as signaling molecules with a diverse range of effects mediated through their action on various nuclear and membrane receptors. In patients with liver disease, total serum BA concentrations are reported to be high; however, BAs are not routinely utilized as biomarkers of choice in any clinical condition.^29^ The myocardium is a very metabolically active organ, and cardiomyocytes have the highest concentration of mitochondria (one-third of the cardiomyocyte volume), the powerhouse for adenosine triphosphate (ATP) generation.^30^ Cardiomyocyte relaxation is an energy-dependent process, requiring removal of cytosolic calcium into the sarcoplasmic reticulum. An ATP deficient state results in impaired cardiomyocyte distensibility, a marker of diastolic dysfunction. Elevated levels of BAs can impair mitochondrial respiration, which adversely influences ATP generating ability.^31^^,^^32^ Animal experiments have demonstrated that elevated BAs lead to transition in mitochondrial substrate utilization from fatty acid to glucose oxidation (fetal mitochondrial metabolism) that is reversible with administration of a BA scavenger (cholestyramine).^33^ In mouse experiments, BA administration resulted in cardiomyocyte hypertrophy; these mice had significantly reduced exercise capacity and achieved lower VO_2_,^34^ findings very similarly observed in these Fontan patients. Moreover, in patients with cirrhosis, total BA was positively associated with left atrial size, suggesting the presence of left ventricular diastolic dysfunction or a high cardiac output state.^35^ BAs can also induce arrhythmias through partial agonistic effect on muscarinic M2 receptors in rat cardiomyocytes.^36^ Cirrhotic cardiomyopathy, characterized by preserved cardiac output and myocardial contractility, but impaired chronotropic response to physiological and pharmacological stimuli, is thought to be due to BA-mediated diastolic dysfunction. The observed blunted peak exercise-augmented heart rate could be also due to elevated BA levels.

### Study limitations

Although bioelectrical impedance analysis technology is inferior to computed tomography and magnetic resonance imaging, it is considered to be on par with the DEXA scan and the consensus guidelines support its use as a reliable, reproducible, portable, and inexpensive technology for body composition measurement.^37^ Due to motion artifact, we could not measure impedance cardiography hemodynamics during different stages of exercise; hence, we obtained resting and immediate postexercise measurements. We also could not conclusively prove cause vs effect between elevated BA and worsening Fontan physiology. However, patients needing establishment of Fontan physiology generally have structurally normal livers at the time of their birth. Hence, liver fibrosis may initiate due to persistently elevated venous pressure and coexisting low cardiac output-induced hepatic ischemia. However, once BAs are elevated, they may potentiate the rate of liver fibrosis as well as myocardial dysfunction; such concepts need to be verified in future research work.

## Conclusions

BAs are elevated in Fontan patients and appear correlated with age, frailty, reduced exercise capacity, and worsened hemodynamic parameters. BAs may be a Fontan-specific biomarker that warrants further exploration.

Perspectives **MEDICAL KNOWLEDGE:** Patients with Fontan circulation are frail, have impaired exercise capability and demonstrate abnormal resting and blunted exercise-augmented hemodynamics, likely the result of an absent subpulmonic ventricle (pre-fill sensitive circulation). These patients also develop progressive liver fibrosis. Fontan-specific biochemical abnormalities that unify the observed multi-organ dysfunction or identify potential therapeutic targets have not been previously described.

**NEW FINDINGS:** Total and selective plasma bile acids (BAs) were markedly elevated in patients with Fontan circulation; and were inversely related to age, frailty, cardio-pulmonary exercise capacity, as well as resting and exercise-associated hemodynamics.

**TRANSLATIONAL OUTLOOK:** This is the first study describing elevated BAs and their relationship with age, degree of frailty, exercise capacity and hemodynamic parameters in Fontan circulation patients. Prior studies have demonstrated BA-mediated mitochondrial dysfunction leading to impaired cardiomyocyte contractility and diastolic dysfunction, impaired response to physiological-pharmacological stress (blunted increase in heart rate and cardiac output), and contribution to progressive liver fibrosis. Future research should focus on 1) exploring and understanding mechanisms underlying BA-mediated cardiomyocyte dysfunction and progressive liver fibrosis, 2) identifying and developing therapies that target BA-mediated cardiomyocyte and/or hepatocyte dysfunction, and 3) exploring BA scavenging therapies aimed at reducing BA levels, which may lead to improved outcomes in this young and extremely high-risk patient population.

## Funding support and author disclosures

This work was supported by Establishment grant—10.13039/100017094Department of Internal Medicine, 10.13039/100010318University of Manitoba, Winnipeg, Canada. The authors have reported that they have no relationships relevant to the contents of this paper to disclose.
