# Supplementary material for: Association of Fontan Pathophysiology With Plasma Bile Acids
Source: JACC Adv. 2025 Jan 18;4(2):101563. doi: 10.1016/j.jacadv.2024.101563 (PMC11787425; doi:10.1016/j.jacadv.2024.101563)
Supplement: Supplementary Material [file mmc1.pdf]

Supplementary material

Non-targeted metabolomic analysis:

Low-molecular-weight metabolites extraction from plasma samples was performed as described previously[1]. One hundred microliters of plasma was mixed with a volume of 200 microliters of acetonitrile. This sample was centrifuged, and supernatant of the mixture was separated for analysed. For accuracy, each plasma sample was aliquoted in duplicate for analysis. Also, a quality control (QC) mixture made of pooled plasma samples were used to validate the extraction and LC-MS methods[2].

The metabolites were analyzed using a 1290 Infinity Agilent HPLC system equipped with a Zorbax Extend-C18 analytical column (2.1 mm × 50 mm, particle size 1.8 μm), (Agilent Technologies, USA). Eluting metabolites were subjected to mass spectral analysis on a 6538 UHD Accurate Q-TOF LC/MS system (Agilent Technologies, CA, USA), controlled via MassHunter Workstation Software (v7.0). Analyses were conducted in both positive and negative mode electrospray ionization (ESI) using a dual ionization source. Raw LC/MS data ('.d files') were preprocessed with Agilent MassHunter Qualitative Analysis (vB.07) and Profinder (vB.06) software. Further data processing and statistical analysis were performed using Agilent Mass Profiler Professional (v12.6), MetaboAnalyst12 software (v3.0, McGill University, Quebec, Canada), MetScape (v3.0, http://metscape.ncibi.org), and Cytoscape (v3.5.1, http://www.cytoscape.org).

References:

1. Mayengbam S, House JD and Aliani M. Investigation of vitamin B(6) inadequacy, induced by exposure to the anti-B(6) factor 1-amino D-proline, on plasma lipophilic metabolites of rats: a metabolomics approach. *Eur J Nutr*. 2016;55:1213-23.
2. Hanson M, Zahradka P, Taylor CG and Aliani M. Identification of urinary metabolites with potential blood pressure-lowering effects in lentil-fed spontaneously hypertensive rats. *Eur J Nutr*. 2018;57:297-308.

Supplementary Table 1: Significantly different bile acids between Fontan patients and healthy subjects

	Fontan (n=20) Mean±SD	Control (n=20) Mean±SD	P-value	
Total bile acid	17601±18084	7163± 5136	0.02	*
DehydroLCA	4.6±4.5	1.2±1.7	0.004	**
LCA	63.7±66.5	19.2±14.2	0.0081	**
7-KetoLCA	64.6±59.4	19.8±11.8	0.0034	**
g-DCA	945.2±939.5	460.7±397.2	0.0435	*
NorCA	17.7±12	8.2±5	0.003	**
UDCA3S	6.5±8.2	2.5±1.6	0.044	*
UDCA3G	54.2±46.4	30.1±9.4	0.0332	*
UCA	13.6±10.9	7.2±3.9	0.0216	*
g-CA	1508.5±1469.2	388.8±347.3	0.0033	**
Glyco-α-Mcholic acid	12.8±10.2	6.5±5.7	0.022	*
g-CDCA3S	425±315.8	141.2±103.2	0.0009	***
t-CA	406.9±719.7	66.1±72.7	0.0483	*
t-CDCA3S	109.3±105.4	38.3±26.5	0.0081	**
g-CA3S	9.4±11	2.5±2.4	0.013	*
g-alloCA3S	2.7±3.9	0.2±0.3	0.0093	**
g-UDCA3S	284.3±417.2	58.9±64.6	0.027	*
t-UDCA3S	15.9±17	3.6±3.3	0.0045	**
Note: p-value <0.05 *, p-value <0.01 **, p-value<0.001 ***				

CA: Cholic acid; CDCA: chenodeoxy cholic acid; DCA: deoxycholic acid; LCA: lithocholic acid; UDCA: ursodeoxy cholic acid

p-value <0.05 *, p-value <0.01 **, p-value<0.001 ***

Supplementary table 2: Bile acid abbreviation, type and polarity (hydrophobicity – hydrophilicity) description

Definition of abbr.

CA, cholic acid
DCA, deoxycholic acid
CDCA, chenodeoxycholic acid
LCA, lithocholic acid
t, Tauro
g, Glyco
MCA, muricholic acid
UCA, ursocholic acid
UDCA, ursodeoxycholic acid
HCA, hyocholic acid
HDCA, hyodeoxycholic acid
G, glucuronide
S, sulfate

Polarity	Compound Group	Suggestedf abbreviation	Ret Time (min)	Type
	Chenodeoxycholic acid	CDCA	14.9	Primary
	Chenodeoxycholic acid-24-glucuronide	CDCA24G	9.3	Primary
	Chenodeoxycholic acid-3-glucuronide	CDCA3G	9.3	Primary
	Chenodeoxycholic acid-3-sulfate	CDCA3S	9.6	Primary
	Cholic acid	CA	11.4	Primary
	Cholic acid-3-sulfate	CA3S	7.8	Primary
	Glycochenodeoxycholic acid	g-CDCA	10.7	Primary
	Glycochenodeoxycholic acid-3-sulfate	g-CDCA3S	6.8	Primary
	Glycocholic acid	g-CA	7.9	Primary
	Glycocholic acid-3-sulfate	g-CA	4.9	Primary

	Glycohyocholic acid	g-HCA	6.8	Primary
	Lamda-Muricholic acid (=hyocholic acid)	λ-MCA	10.5	Primary
	Taurochenodeoxycholic acid	t-CDCA	8.8	Primary
	Taurochenodeoxycholic acid-3-sulfate	t-CDCA3S	5.2	Primary
	Taurocholic acid	t-CA	6.8	Primary
	Taurohyocholic acid	t-HCA	5.8	Primary
	3β,7α-Dihydroxycholestenoic acid	3β,7α-Dihydroxycholestenoic acid	16.7	Primary bile acid synthesis intermediate
Most Hydrophobic	3β-Hydroxy-5-cholestenoic acid	3β-Hydroxy-5-cholestenoic acid	20.6	Primary bile acid synthesis intermediate
	7α-hydroxy-3-oxo-4-cholestenoic acid	7α-hydroxy-3-oxo-4-cholestenoic acid	17.2	Primary bile acid synthesis intermediate
	3α,7α-Dihydroxycholestanoic acid	DHCA	16.6	Primary bile acid synthesis intermediate
	3α,7α,12α-Trihydroxycholestanoic acid	THCA	16.4	Primary bile acid synthesis intermediate
	α-Muricholic acid	α-MCA	9.2	Primary in mouse
	β-Muricholic acid	β-MCA	9.6	Primary in mouse
	Tauro-α-muricholic acid	t-α-MCA	4.7	Primary in mouse
	Tauro-β-muricholic acid	t-β-MCA	4.9	Primary in mouse
	12-Ketochenodeoxycholic acid	12-KetoCDCA	10.2	Secondary
	12-Ketolithocholic acid	12-KetoLCA	13.4	Secondary
	3-Oxocholic acid	3-OxoCA	11.1	Secondary
	6,7-Diketolithocholic acid	6,7-DiketoLCA	13.1	Secondary
	7-Ketodeoxycholic acid	7-KetoDCA	9.6	Secondary
	7-Ketolithocholic acid	7-KetoLCA	13	Secondary
	Allocholic acid	AlloCA	11.2	Secondary
	Allocholic acid-3-sulfate	AlloCA3S	7.5	Secondary
	Alloisolithocholic acid	AlloisoLCA	17.2	Secondary
	Apocholic acid	ApoCA	13.5	Secondary
	Dehydrocholic acid	DehydroCA	8.5	Secondary
	Dehydrolithocholic acid	DehydroLCA	20.2	Secondary

	Deoxycholic acid	DCA	15.4	Secondary
	Deoxycholic acid-24-glucuronide	DCA24G	9.8	Secondary
	Deoxycholic acid-3-glucuronide	DCA3G	9.5	Secondary
	Deoxycholic acid-3-sulfate	DCA3S	10	Secondary
	Dioxolithocholic acid	DioxoLCA	8.1	Secondary
	Glycoallocholic acid	g-alloCA	7.6	Secondary
	Glycoallocholic acid-3-sulfate	g-alloCA3S	4.6	Secondary
	Glycodehydrocholic acid	g-dehydroCA	4.5	Secondary
	Glycodeoxycholic acid	g-DCA	11.3	Secondary
	Glycodeoxycholic acid-3sulfate	g-DCA3S	7	Secondary
	Glycohyodeoxycholic acid	g-HDCA	7.9	Secondary
	Glycohyodeoxycholic acid-3-sulfate	g-HDCA3S	4.6	Secondary
	Glycolithocholic acid	g-LCA	15	Secondary
	Glycolithocholic acid-3-sulfate	g-LCA3S	9	Secondary
	Glycoursodeoxycholic acid	g-UDCA	7.7	Secondary
	Glycoursodeoxycholic acid-3-sulfate	g-UDCA3S	4.4	Secondary
	Hyodeoxycholic acid	HDCA	11.9	Secondary
	Isodeoxycholic acid	IsoDCA	18.5	Secondary
	Isolithocholic acid	IsoLCA	18.2	Secondary
	Isolithocholic acid-3-sulfate	IsoLCA3S	11	Secondary
	Lithocholic acid	LCA	19.6	Secondary
	lithocholic acid-24-glucuronide	LCA24G	12.7	Secondary
	lithocholic acid-3-glucuronide	LCA3G	12.3	Secondary
	lithocholic acid-3-sulfate	LCA3S	12.2	Secondary
	Murocholic acid	Murocholic acid	10.8	Secondary
	Norcholic acid	NorCA	9.4	Secondary
	Nordeoxycholic acid	NorDCA	9.5	Secondary
	Norursodeoxycholic acid	NorUDCA	13.4	Secondary

	omega-Muricholic acid	ω-MCA	8.9	Secondary
	Tauroallocholic acid	t-alloCA	6.6	Secondary
	Taurodehydrocholic acid	t-dehydroCA	3.9	Secondary
	Taurodeoxycholic acid	t-DCA	9.3	Secondary
	Taurodeoxycholic acid-3-sulfate	t-DCA3S	5.4	Secondary
	Taurolithocholic acid	t-LCA	11.7	Secondary
	Taurolithocholic acid-3-sulfate	t-LCA3S	6.8	Secondary
	Tauro-omega-muricholic acid	t-ω-MCA	4.4	Secondary
Most hydrophilic	Tauroursodeoxycholic acid-3-sulfate	t-UDCA3S	3.4	Secondary
	Tauroursodexycholic acid/Taurohyodeoxycholic acid	t-UDCA/t-HDCA	6.6	Secondary
	Ursocholic acid	UCA	7.9	Secondary
	Ursodeoxycholic acid	UDCA	11.7	Secondary
	ursodeoxycholic acid-24-glucuronide	UDCA24G	8.7	Secondary
	ursodeoxycholic acid-3-glucuronide	UDCA3G	8.3	Secondary
	ursodeoxycholic acid-3-sulfate	UDCA3S	8.6	Secondary
